# The Comprehensive Health Risk Assessment of Polish Smelters with Ecotoxicological Studies

**DOI:** 10.3390/ijerph191912634

**Published:** 2022-10-03

**Authors:** Magdalena Wróbel, Agnieszka Trzyna, Farhad Zeynalli, Justyna Rybak

**Affiliations:** Faculty of Environmental Engineering, Wroclaw University of Science and Technology, Wybrzeże Wyspiańskiego 27, 50-370 Wrocław, Poland

**Keywords:** heavy metals, soil, smelter, health risk, environmental risk assessment

## Abstract

Air pollution connected to smelter activity can significantly deteriorate the quality of soil due to the precipitation of rain or simple deposition of the air particulates into the ground. Hence, in this study, we focused on the analysis of the soil which can inform us about the general state of the environment in the area and the possible health hazard for humans. If the top layer of the soil is in bad condition, it can indicate that air pollution in the area is also not in good condition, and the lives of the inhabitants of these areas can be at serious risk. To comprehensively identify the level of contamination in the soils from the areas of Polish smelters, studies of the concentration of potentially toxic elements (PTEs) in the soil were conducted. On the basis of the obtained results, health risk assessment was performed to verify the possible influence on human health. The results showed that the non-carcinogenic risk existed only for Oława, while the possibility of the carcinogenic risk occurred in all of the studied places. The outcome is very disturbing and certain steps must be undertaken in order to protect the lives of the inhabitants. Additionally, in order to assess the suitability of soils for the cultivation of edible plants, phytotoxicity tests were conducted. The obtained results revealed that in all the studied areas, a visible inhibition of roots and shoots can be observed. The conducted study indicated the most polluted regions and the possible health hazard, and it can provide the general information about the impact of smelters on the environment.

## 1. Introduction

Soil is the biologically active top layer of the earth’s crust. It is considered to be a main part of biogeochemical system, playing an important role in retention, storage, and cycling of elements [1,2]. One of the most essential functions of soil is being a reservoir of chemical elements necessary for life and development of plants and living organisms [3]. Some of these elements can be included in the group of heavy metals. Although some of them are essential for life, they can be also very harmful to humans, animals, plants, and microorganisms if present at toxic levels. Occurrence of these heavy metals in soil is mainly connected with natural weathering of parent rock but also with anthropogenic activities, i.e., mining or industrial processes [4,5].

The processes of metal mining and smelting are strictly connected with the emissions of different pollutants. The produced pollution is often characterized by elevated concentrations of potentially toxic elements (PTEs) which are known to be very harmful to environment, as well as human health, according to their toxicity, persistence, and biological accumulation [6,7]. Smelting processes of metals introduce to the atmosphere such elements in the form of dust particles which subsequently are deposited on the soil surface. PTEs emitted from mining operations are mostly found in the fine particulate matter (PM) fraction (<2 μm) while those from smelting are concentrated in the ultrafine fraction (<0.5 μm). According to this fact, they can travel long distances and an elevated accumulation of PTEs can occur, especially in the area covered by predominant wind direction [2,8]. PTEs accumulate in soil gradually and because of their persistence, their residence time is quite long. Moreover, the purification of soils contaminated with PTEs via natural processes is very slow [2]. Many factors can influence the distribution of PTEs in soil, i.e., the mineralogy of soil and PM, chemistry of emitted dust, and the environmental variables. These environmental variables include pH and organic matter content. [2]. The smelting of metal ores is one of the most important sources of environmental pollution by metals and metalloids. Soil pollution can also originate from dissolution of smelter wastes which can lead to a serious soil contamination, noted even for a long time after the closure of the smelter [9]. Metal mining and processing is responsible for global Hg, Cd, and Pb emissions [8]. Due to the installation of flue gas cleaning systems, the emissions from the metal industry decreased in Europe over the last 60 years [10]. Despite such solutions, contaminants are still present in the soil as it represents direct sinks for pollutants emitted by smelters. This, in turn, can cause serious effects for soil ecosystems and can have a negative impact on biological processes and health of living organisms [11,12]. Moreover, soil contamination influences the growth of plants to different extents, depending on the presence of various heavy metals, their concentration, chemical form, plant species, and soil composition. [13,14]. Hence, as a result of soil contamination we can observe inhibition of root and shoot growth, affection of productivity, or lower quality crops. Considering this, it is clear that soil contamination can affect the food safety at the very beginning of a food chain, which is known to be one of the greatest threats to human health [15]. 

The biggest deposits of copper in Europe occur in the Legnica-Głogów Copper District (Lower Silesia in Poland) [16]. The Głogów Copper Smelter has two process lines—Głogów I and Głogów II—which use single-stage flash smelting of copper concentrates directly to blister copper with an approximate 98.5% Cu content. The technology of the Legnica Copper Smelter is based on smelting of copper concentrate in shaft furnaces. The primary product is electrolytic copper in the form of cathodes (https://kghm.com/en/our-business/metallurgy-and-refinery, accessed on 21 July 2022). However, despite the fact that many changes in technology of both smelters were introduced, which improved the quality of the environment in the area, the ecological damage that occurred in the initial years of the smelters operation still persists. Particularly, this refers to soil in which heavy metals have bioaccumulated [17].

The smelter “Oława” located in Oława (Lower Silesia) is a branch of the company Z.M. SILESIA S.A. The history of the company dates back to the mid-nineteenth century. The first information about the zinc white produced in Oława comes from 1845. This smelter is the largest producer of zinc, lead, and cadmium oxides in Poland. Zinc white is produced with the condensation method, which involves the melting of electrolytic zinc (99.95% Zn). The products are used in the rubber, ceramics, and pharmaceutical industries. They manufacture components necessary for the production of batteries and accumulators, as well as compound feed, paints, and varnishes [18]. Although the contaminated areas are successively recultivated there is still a potential risk of the presence of these pollutants in the soil. This is due to the fact that degraded land reclamation strategies in Poland are based on the immobilization of pollution and the development of the land surface, not on removing pollution, which is related to technical and economic reasons [19].

The heap in Siechnice (Lower Silesia) is a remnant of the chrome steel smelter operating until 1995. The smelter was closed after protests by the inhabitants of Wrocław—the main reason for the protests was the proximity of aquifers. 

This paper’s aim is to summarize the knowledge of the possible impact of smelters on soils in their vicinity and define a probable health risk to inhabitants of the Lower Silesia region (Poland). Additionally, where possible, studies of air pollution in these cities were found in order to check whether the most commonly occurring PTEs in the air are in accordance with those found by us in the studied soil. We aimed to present a complex study of possible soil contamination by determining selected PTEs: Hg, Cu, and As in the Głogów and Legnica Copper Smelters; Zn, Cu, Pb, Fe, and Cr in Siechnice; and Zn, Pb, and Cr in the Smelter “Oława”. Moreover, a comparison of the phytotoxicity of soils from the Głogów and Legnica Copper Smelters, Siechnice, and Smelter “Oława” was carried out in order to assess the suitability of soils for the cultivation of edible plants. To understand the possible environmental risk and, therefore, potential risk to human health, the following indices were calculated—average daily dose (ADD), hazard quotient (HQ), hazard index (HI), and excessive risk of developing cancer (ECR)—to get a complex view of the impact of smelters on human health of inhabitants and to assess utility of these indices for the comprehensive evaluation of possible health risk.

## 2. Materials and Methods

### 2.1. Study Area

**Figure 1:**
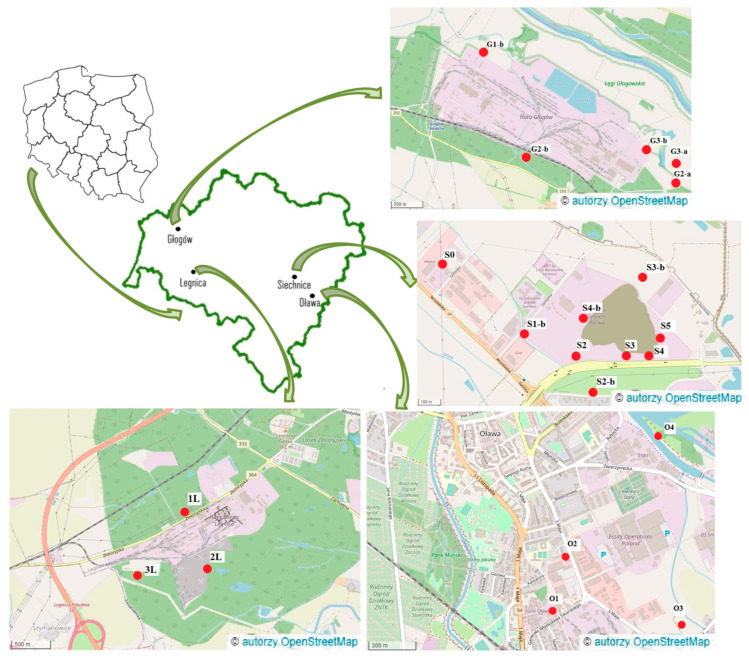
Studied smelters with sampling points on the map of Lower Silesia. Adapted with permission from OpenStreetMap [OpenStreetMap contributors. (2015) Planet dump (Data file from $date of database dump$). Retrieved from https://planet.openstreetmap.org/]. 2015, OpenStreetMap (accessed on 27 September 2022).

Figure 1 shows the area of study, i.e., smelters and sampling points. The detailed description of sample collection sites is presented in Table S1 (Supplementary Material).

#### 2.1.1. Siechnice

The urban–rural municipality of Siechnice is a city characterized by the post-industrial areas created after the closure of the of Siechnice smelter. In 1910, the power plant and carbide factory were built there. The carbide production was reduced, and ferroalloys and welding powders became the main products in 1932. Later, the factory was transformed into the smelter of ferroalloys (for military targets) and named “Siechnice”. However, due to the protests of the inhabitants of Wrocław and Siechnice in 1989–1999 connected with the unfavorable location of the smelter, the smelter was finally closed. A large heap of post-smelting slag (composed of ashes and production waste) located in the north-western part of the city was left, which still raises concerns among local residents. This iron–chromium heap is suspected of having a negative impact on the nearby environment, especially on the soil and groundwater. The PTEs leach from there and also reach the nearby soil from which they can be subsequently leached, leading to the contamination of the water. As a result, an elevated concentration of Fe, Pb, and Cr were recorded in soil and groundwater. Moreover, the highest concentrations of the above-mentioned elements were recorded in plants [18].

#### 2.1.2. Głogów

The other industry is the Głogów Copper Smelter which was established in 1971 and belongs to the KGHM Polska Miedź S. A. company. The quality of the air had been also a serious threat for many years there (as a result of emissions of SO_2_, NO_2_, PM, and heavy metals). Finally, after 1990, due to limitations presented by Ministry of the Environment, Natural Resources and Forestry, the emission of harmful substances was significantly reduced [20], although scientists proved that the limits of the copper and lead concentrations in soil are still exceeded in the vicinity of smelter [21]. Moreover, the smelter is located in the vicinity of the urban agglomerations and agricultural areas where crops (mainly wheat, potatoes, and sugar beets) are cultivated. The problem is that all smelter activities connected with the extraction and processing of copper ores may have a significant hazardous effect on the environment [17].

#### 2.1.3. Oława

The smelter “Oława” is a part of metallurgical plants, “Silesia”, located in the city of Oława. The smelter began its activity in 1845 and produced different forms of zinc white and lead oxide. Currently, the smelter produces zinc white as well as lead oxide and cadmium oxide [19]. In the 1980s, the Oława Smelter was listed as one of the largest polluters in Poland, but in 1994, with a decision of the Main Inspectorate for Environmental Protection (GIOS), it was removed from the list. Currently, modern technologies applied in the smelter are efficient and successfully reduce emitted pollution; however, the contamination of sediments or soil still persist [22].

#### 2.1.4. Legnica

The Legnica Copper Smelter also belongs to the KGHM Polska Miedź S. A. company and produces mainly electrolytic copper in the form of cathodes and refined silver. Additionally, gold, lead, palladium, platinum concentrate, and rare earth elements in smaller quantities are produced there. It was opened in 1953. Primarily, it emitted fly ash characterized by high amounts of PTEs, but in the 1980s and 1990s, the level of pollutants was significantly reduced (Monograph of KGHM Polish Copper Company 2007). However, even now the smelter is known to be one of the biggest sources of pollution for inhabitants. There is a problem of air and soil contamination [23,24]. Furthermore, the reports issued by Provincial Inspectorate for Environmental Protection (WIOS) also demonstrated the problem mainly with the air pollution in this area (especially with arsenic and PAHs) [25]. 

### 2.2. Methods

Soil samples were collected according to the Polish norm [PN-R04031:1997] in four different cities, all connected with present (Legnica, Głogów, and Oława) or past (Siechnice) smelter activities. The soil samples (1 kg) were a mixture of soil taken from 20 and 40 cm of the arable layer at 21 sites. At each site, soils were randomly sampled at 4–6 locations and mixed together to create a composite sample. The control samples, on the other hand, were collected far from the source of contamination (several kilometers from the examined smelters). The samples were dried and sieved to remove larger contaminants in accordance with the PN-ISO 11464 standard, and the pH of soil samples was measured in accordance with the PN-ISO 10390 standard. Then, samples were weighed, milled, and mineralized (PTEs assessment) or used in the ecotoxicological tests. The estimation of the dry mass was performed based on the norm PN-EN 14346:2011.

#### 2.2.1. PTEs Analyses

In the samples taken near the Głogów and Legnica Copper Smelters, Cu, Hg, and As concentrations were assessed; in the soil from Siechnice, Zn, Cu, Pb, Fe, and Cr were assessed; and Zn, Pb, and Cr near Smelter “Oława” were assessed. Different elements were chosen due to the different characteristics of the deposits present there. According to The International Agency for Research on Cancer (IRAC), the above-mentioned elements are classified as toxic to human health. In the mineralization process, an equality of 8 mL 65% high purity nitric acid was used. The acid was introduced into each sample and, with the use of MILESTONE START D Microwave Mineraliser, the samples were digested. The process was based on four stages with different times and temperatures, and it lasted 32 min in total. After the end of the process and the cooling of the samples, the vessels were rinsed with deionized water. Then, the samples were filtered into 50 mL graduated flasks using a hard, quantitative paper filter. Along with water extracts and digests of soil samples, an appropriate blank sample was prepared. Blank samples were analysed together with other samples. Element concentrations in blank samples were subtracted from the concentrations obtained for studied samples. The concentrations of Cu, Zn, As, Pb Cr, and Hg in water extracts and digests were assessed with ICP-MS (Elan 6100 DRC-e Perkin Elmer). The operating conditions were: ICP RF power: 1125 W; nebulizer gas flow rate: 0.78–0.83 L/min; auxiliary gasflow: 1.15 L/min; plasma gasflow: 15 L/min; and sample flow rate: 1 mL/min. The samples were analysed in triplicate. As calibration solutions were certified multi-element standard stock solutions, Periodic table mix 1 and Transition metal mix 2 (Fluka) were applied. The validation was carried out with two certified reference materials, SRM 1648a (recovery in the range 75 (As)–127% (Cr)) which was obtained from the National Institute of Standard and Technology (NIST). Detection limits were: 0.151 µg/l for Zn; 0.019 µg/L for As; 0.013 µg/L for Cr; 0.022 µg/L for Pb; 0.048 µg/L for Cu; and 0.014 µg/L for Hg.

#### 2.2.2. Phytotoxicity Assessment 

The first stage of phytotoxicity tests was to assess the germination capacity of seeds; for this purpose, 50 seeds of representatives of the monocots: corn (*Zea mays*), wheat (*Triticum aestivum*), and dicots: lupinus (*Lupinus luteus*) were placed in Petri dishes without access to lights, with constant humidity, and at a temperature of 20 °C. The germination tests were performed in 3 replicates. The ability to germinate seeds for each plant was high (over 90%). The vase test (phytotoxicity test) consisted of 20 germinated seeds of previously chosen species (*Zea mays, Triticum aestivum*, and *Lupinus luteus*). The following dilutions of contaminated soil mixed with the control soil were prepared: 100%, 50%, 25%, 12.5%, and 6.25%. Throughout the experiment, constant humidity, i.e., 80% WHC (water-holding capacity), and lighting were maintained for 16 h per day. After 14 days, the lengths of shoots and roots of each specimen were measured. The test was performed in accordance with the PN-EN ISO 11269-2: 2013-06. Phytotoxicity of the studied metal on root and shoot elongation was studied. 

## 3. Health Risk Assessment 

### 3.1. Exposure Dose

Health risk assessment was carried out using a method compliant with the US EPA [26]. The evaluation shows the exposure to elements such as Zn, Cr, Cu, Fe, Pb, Cd, As, and Hg. The calculations included both the exposure of an adult and a child throughout their lives, and was divided into three routes of entry: oral, inhalation, and dermal. Health exposure was estimated based on the amount of harmful substance with which the organism cools down during the whole day, per 1 kg of body weight. Calculations were carried out using the following formulas:

$$\mathrm{ADDing}=C*\frac{\mathrm{IngR}*\mathrm{EF}*\mathrm{ED}}{\mathrm{BW}*\mathrm{AT}}$$

$$\mathrm{ADDinh}=C*\frac{\mathrm{InhR}*\mathrm{EF}*\mathrm{ED}}{\mathrm{PEF}*\mathrm{BW}*\mathrm{AT}}$$

$$\mathrm{ADDderm}=C*\frac{\mathrm{SL}*\mathrm{SA}*\mathrm{ABS}*\mathrm{EF}*\mathrm{ED}}{\mathrm{BW}*\mathrm{AT}}$$

where:

C—average metal concentration in soil [mg·kg^−1^];
IngR—value of daily accidental soil intake [mg/d];
InhR—daily lung ventilation [m^3^/d];
EF—contact frequency [d/year];
ED—duration of contact [year];
BW—average body weight [kg];
AT—averaging period [d];
PEF—particle emission factor [m^3^/kg];
SL—coefficient of soil adherence to the skin [mg/cm^2^·d];
SA—skin surface exposed to soil [cm^2^];
ABS—percutaneous absorption coefficient, unnamed quantity [27].

The values for the above parameters for children and adults are shown in Table S2, Supplementary Material. 

### 3.2. Non-Cancerogenic Health Risk

Parameters such as Hazard Quotient (HQ) and Hazard Index (HI) can be used to assess health risk. These equations are as follows:

The amount of hazard (HQ) and the hazard index (HI) are the parameters that allow the assessment of health risk and are calculated according to the following formulas:

$$\mathrm{HQ}=\frac{\mathrm{ADD}}{\mathrm{RfD}}$$

HI=∑HQ

where ADDing, ADDinh, ADDderm are the ingestion, inhalation, or dermal dose, respectively. In contrast, RfD is the reference dose that is given in the Integrated Information Risk System (IRIS) [27]. Table S3 in Supplementary Material shows the values for RfD for different elements. If HQ is larger than 1, adverse effects on human health can occur, while for HQ < 1, there is no risk. HI is measured similarly; if HI < 1, there is no risk of health hazards [26].

### 3.3. Risk Calculation

The excessive risk of developing cancer (ECR) is calculated using the formula:

$$\mathrm{ECR}=\frac{C*\mathrm{ET}*\mathrm{EF}*\mathrm{ED}*\mathrm{IUR}}{\mathrm{BW}*\mathrm{AT}}$$

However, it should be remembered that it is not possible to use all the metals tested for this assessment, as not all of them are carcinogenic. Thus, the elements Pb, Cr, Cd, and As were used to calculate the ECR. The IUR values of Pb, As, Cd, and Cr are 1.2 × 10^−5^, 4.3 × 10^−3^, 1.8 × 10^−3^, and 0.012 (g/m^3^)^−^^1^, respectively (IRIS). The ET value means exposure time and is 8 h/d for children and 16 h/d for adults, respectively. If the ECR ranges is 10^−6^–10^−4^, there is a low risk of cancer [27].

## 4. Statistical Analysis

The data (the concentrations of metals) were processed by statistical tests using STATISTICA^®,^, Wrocław, Poland. They were checked for normal distribution (Shapiro–Wilk W test) and homogeneity of variance (Levene’s test). Tests of significance were made at the 95% confidence level. The significance of differences (concentrations of studied metals) among sites within every smelter were performed by one-way ANOVA.

For pyhotoxicity studies, IC50 (inhibition concentration) was applied, which is a statistically calculated concentration that gives an effect in an environmental medium in 50% of the tested plants species. Inhibition concentration is used for effects other than death of studied plants. This refers to the concentration which causes a 50% inhibition of root and shoot elongation of studied plants. A higher value of IC50 means less toxicity. The logistic regression model was applied to establish IC50.

## 5. Results

### 5.1. Soil Contamination 

In the past, the emission of harmful substances occurred on a large scale and now their impact occurs in the form of soil pollution. The concentrations of selected metals were assessed in studied areas (Table 1). Significant differences (*p* values < 0.05) among samples collected at different sites within every studied smelter are presented in Table S4 (Supplementary Material). The concentrations of Zn, Pb, and Cd recorded in Oława (O1 and O2) were above the permissible limits according to the Regulation of the Minister of the Environment [ROZPORZĄDZENIE MINISTRA ŚRODOWISKA z dnia 1 września 2016 r] (Table S5, Supplementary Material). The highest concentration of Zn was found at O1 (2411.93 ± 27.16 mg·kg^−1^) in the “Oława” Smelter, while the lowest values were observed at S2 (44.49 ± 17.81 mg·kg^−1^) in Siechnice. The highest concentration of Pb was found at O1 (1903.02 ± 48.13 mg·kg^−1^) in the “Oława” Smelter, while the lowest values were observed at S2 (13.13 ± 0.14 mg·kg^−1^) in Siechnice. Cd was assessed only in the “Oława” Smelter, and the highest value was at O1 (4.97 ± 0.71 mg·kg^−1^). Cu content at all sites exceeded threshold values. The highest concentration was recorded at G2-a (1316.78 ± 44.26 mg·kg^−1^) and the lowest at S2 (73.01 ± 7.1 mg·kg^−1^). The highest level of Fe was found at S3 (13,440.62 ± 342.63 mg·kg^−1^) in Siechnice, which is related directly to the profile of former smelter activity. The comparison of Oława and Siechnice indicated higher contamination with Zn and Pb in the “Oława” Smelter. However, in Siechnice, at S0 and S5, Cr values were approximately 2.3 times higher than the allowable levels (696.30 ± 21.14 mg·kg^−1^ at S0 and 705.31 ± 29.884 mg·kg^−1^ at S5, respectively). Hg concentration exceeded the limit value for soil only in Legnica, L1 (4.07 ± 0.60 mg·kg^−1^). The concentration of studied elements in both the Głogów and Legnica Copper Smelters were in the following order: Cu > As >Hg, with Głogów being more polluted with Cu. 

### 5.2. Phytotoxicity

**Figure 2:**
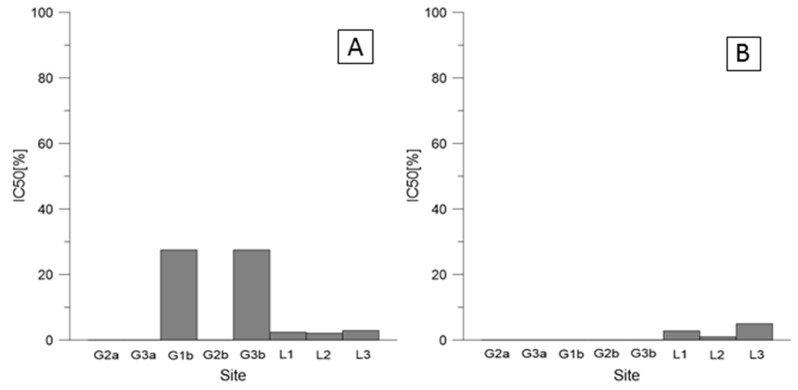

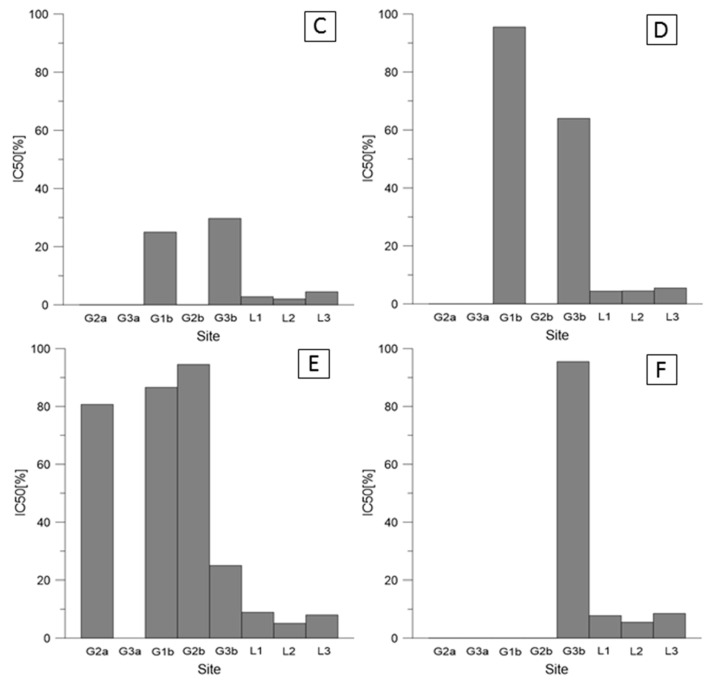
IC50 [%] values for soil at 100% concentration (Głogów and Legnica): (**A**) roots of *L. luteus*, (**B**) shoots of *L. luteus*, (**C**) roots of *T. aestivum,* (**D**) shoots of *T. aestivum,* (**E**) roots of *Z. mays,* and (**F**) shoots of *Z. mays*.

Figure 2 shows the IC50 values for root and shoot length inhibition for selected species in the Głogów and Legnica Copper Smelters. The values varied. The roots, due to their function and direct contact with the soil, are usually more sensitive to toxic effects than the shoots, as evidenced by the obtained results. For instance, in Głogów, maize roots (*Z. mays*) were found to be the most sensitive to soil contamination (even more than its shoots), while lupine (*L. luteus)* was the least sensitive (Figure 2). In general, lower values for all plants were obtained for the Legnica Copper Smelter which suggest the higher impact of this smelter on the environment.

**Figure 3:**
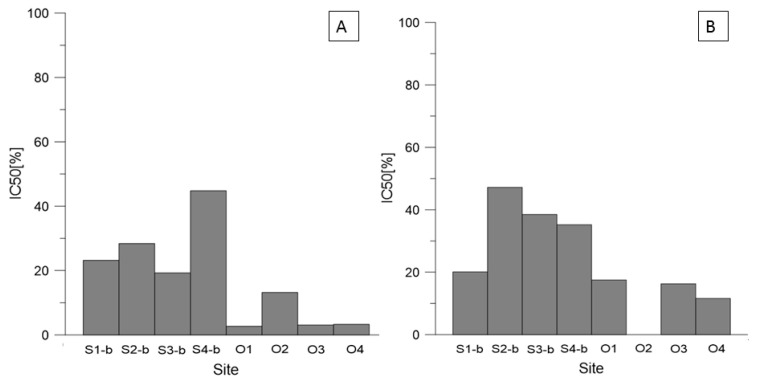
IC50 [%] values for soil at 100% concentration (Siechnice and Oława): (**A**) roots of *A. sativa* and (**B**) shoots of *A. sativa*.

A negative influence of the soil contamination on the growth of roots and shoots was observed not only in Głogów and Legnica but also in Oława and Siechnice. Figure 3 presents IC values for these smelters. In this case, the studied plant species was *A. sativa*. The lowest IC50 values were obtained in Oława (3.14% and 3.19% for roots growth of *A. sativa)*, suggesting high toxic effect of soil in this area. In Siechnice, the highest inhibition of the root length was observed in the sample from S-3b (19.3%). 

### 5.3. Health Risk Assessment

#### 5.3.1. Average Daily Dose (ADD), Hazard Quotient (HQ), and Hazard Index (HI)

Health exposure is determined by analysing three parameters: average daily dose (ADD), Hazard Quotient (HQ), and Hazard Index (HI). The values of these indicators for each of the studied smelters are presented in the supplementary material (Tables S6–S12). The highest ADD values for the Legnica Copper Smelter were observed at point L1 for children, especially in the case of Cu (ADDing = 3.40 × 10^3^ mg·kg^−1^) and Hg (ADDing = 1.34 × 10^1^ mg·kg^−1^). The lowest ADD values for this area were noticed at point L3 for adults exposed to Hg contamination (ADDinh = 4.76 × 10^−5^ mg·kg^−1^) (Table S6). Analysing the HQ and HI values for the Legnica Copper Smelter (Table S7), it can be seen that none of the given values were >1; hence, no health risk was observed. The highest values for these indicators were noted at point L1 (in the case of Cu for children, HQ was equal to 8.51 × 10^−2^, and HI was equal to 8.67 × 10^−2^).

The results of health exposure indicators for the Głogów Copper Smelter are shown in Table S8. The highest ADD value was recorded at point G2-a for Cu in the case of ingestion of the pollution (ADDing = 4.33 × 10^3^ mg·kg^−1^ for children). The lowest dose was also noted at point G2-a, but for Hg for the respiratory tract (ADDinh = 4.49 × 10^−5^ mg·kg^−1^ for adults) (Table S8). None of the examined points of the Głogów Copper Smelter HQ and HI indices exceeded 1; therefore no health risk was found.

The ADD values for the Oława Smelter are shown in Table S9, and HQ and HI values are shown in Table S10. The highest ADD value was recorded at point O1 for Zn (ADDing = 7.89 × 10^3^ mg·kg^−1^ for children). In addition, a high value was noted in this point in the case of Pb (ADDing = 6.24 × 10^3^ mg·kg^−1^ for children). The lowest dose was obtained at point O4 for Cd (ADDinh = 2.03 × 10^−5^ mg·kg^−1^ for adults). In this study area, HQ and HI values ranged between 1.2 × 10^−8^ and 4.53 × 10^0^. All the values greater than 1 suggest that a risk to human health exists. Such high values were noted for the point O1 for Pb (HQing = 4.46 × 10^0^ for children and HQing = 1.91 × 10^0^ for adults, HI = 4.53 × 10^0^ for children, and HI = 2.01 × 10^0^ for adults). Another sampling point with high values was point O2, reaching 1.01 × 10^0^ in the case of HQing and 1.03 × 10^0^ in the case of HI for children for Pb. The lowest values of HQ and HI were obtained for the point O4 (HQinh = 6.76 × 10^−9^, HI = 1.88 × 10^−4^) for Zn for adults (Table S10).

Very high values of ADD were recorded at points located close to the smelter “Siechnice” (Table S11). The highest values of all tested metals for this smelter were recorded for Fe, ranging between 2.11 × 10^4^ mg·kg^−1^ and 4.42 × 10^4^ mg·kg^−1^ for exposure to pollution by ingestion. The highest value was calculated for point S3 and the lowest for point S4. For Zn, the highest value was recorded at point S0 (ADDing = 2.79 × 10^2^ mg·kg^−1^) while for Cu, Cr, and Pb, the highest value was at point S5 (ADDing = 2.91 × 10^2^ mg·kg^−1^, ADDing = 2.32 × 10^3^ mg·kg^−1^, and ADDing = 1.46 × 10^2^ mg·kg^−1^, respectively). All of the highest values were noted for children’s exposure to pollution by ingestion. On the other hand, most of the lowest ADD values were observed for adults. For instance, for Zn and Fe, the lowest values were at point S4 (ADDinh = 4.51 × 10^−3^ mg·kg^−1^ and ADDinh = 6.5 × 10^−1^ mg·kg^−1^, respectively), for Cu and Pb in point S2 (ADDinh = 7.40 × 10^−3^ mg·kg^−1^ and ADDinh = 1.33 × 10^−3^ mg·kg^−1^, respectively), and for Cr in point S3 (ADDinh = 3.16 × 10^−3^ mg·kg^−1^). Table S12 shows the values of HQ and HI indices for each of the examined elements for the Siechnice smelter. The highest values for HQ and HI were recorded at point S5 for Cr (HQing = 7.73 × 10^−1^ and HI = 7.82 × 10^−1^, both for children) while the lowest values were recorded at point S4 for Zn (HQing = 1.50 × 10^−8^ and HI = 2.30 × 10^−4^, both for adults). All the calculated HQ and HI values for the Siechnice smelter were not greater than 1; therefore, it can be concluded that the health risk did not occur (Table S12). 

#### 5.3.2. Excess Cancer Risk (ECR)

An excessive risk of cancer (ECR) is considered when the ECR values are greater than 10^−4^ (Table 2). Such high values were recorded for the examined points in the Siechnice smelter, where the highest ECR value for Cr for children was at the point S5 is 2.23 × 10^0^. A similar high value was calculated for the S0 point (ECR = 2.2 × 10^0^ for Cr for children). For each examined point in Siechnice, ECR values for Cr were high; hence, it can be concluded a high risk of cancer exists. Lower values were noted for Pb, for which the maximum ECR value was 9.32 × 10^−4^ for children at the point S5. It follows that the Siechnice Smelter can be harmful to health when considering Cr and Pb contamination. When analysing the Głogów Smelter, the values of ECR are lower than in the Siechnice Smelter, but still the risk can be considered high (ECR = 7.07 × 10^−2^ for As for children and ECR = 2.65 × 10^−2^ for As for adults). For the “Oława” Smelter, the ECR values are greater than 10^−4^, which indicated a high risk of cancer. The highest value was recorded at point O1 (ECR = 4 × 10^−2^ for Pb for children), and the lowest at point O4 (ECR = 2 × 10^−4^ for Pb for adults). ECR values for the Legnica Smelter for As varied between 4.33 × 10^−3^ and 8.60 × 10^−2^, which also indicated a high risk of cancer (Table 2).

## 6. Discussion

### 6.1. Soil Contamination

Mining and ore processing cause many potential environmental risks, and among them, the most important are ore processing waste production and deposition of atmospheric particles from smelters. The important receptors are soils, which accumulate toxic metals. The location is important as it influences the size of impact of a smelter on the environment. Pollutants released by smelter activities can have negative effects on the ecosystem and human life. Most commonly, the negative effects on vegetation are registered [28]; metal uptake by plants, especially crops and other edible vegetables can be very dangerous [29]. In addition, elevated levels of metals in human blood and urine are a common impact of smelter neighbourhood [30]. Among our results (Table 3), the worst impact on the environment in terms of As contamination was the Głogów Copper Smelter, although compared with PTE concentrations in other regions of the world, especially China [31], this value noted by us is rather low. According to the fact that Cd contamination study was restricted only to the “Oława” Smelter, we cannot compare the obtained values to other studies sites, but it can be noticed that with other regions of the world, the impact of the “Oława” Smelter is rather small. Again, Cr contamination assessment was done in Oława, but contrary to the previous results, the concentration of Cr is surprisingly high. In the case of Cu, the highest values were recorded at the Głogów Copper Smelter which was relatively high among other values recorded worldwide. Pb concentration was quite high in the “Oława“ Smelter, similar to the other study conducted in Poland [32]. Contamination by Zn is not high when we compared it with other sites. Regarding Fe, a high value was recorded in Siechnice, which is not surprising because the former smelter used old technologies and iron processing was not effective enough; hence, the high soil contamination with this metal occurs in the smelter surroundings. The values of Hg have been studied in the Legnica and Głogów Copper Smelters only. The highest values have been obtained in Legnica, which is in line with other findings, indicating that the impact on environment of this smelter is higher than Głogów.

### 6.2. Phytotoxicity 

Even though the PTEs are naturally present in the soil and the plants need some of them for their growth, the excessive amounts can become toxic to plants. When the optimal levels are exceeded, the adverse effects on the plants can be observed [37]. In the areas of the Legnica and Głogów Copper Smelters, the amount of Cu in the soil clearly exceeded the permissible concentration (Table 3, Table S5). Elevated copper content in the soil impedes shoot growth and changes the structure of the root system [38,39,40], which was confirmed by the IC50 results in Głogów and Legnica where maize (*Z. mays*) was the most sensitive species (Figure 2). The study showed that in the area of the Legnica Copper Smelter, higher toxicity on plants was observed when compared with Głogów, which indicates a bigger impact of this smelter on the environment. This might be connected mainly to a higher Hg amount found in the soil in Legnica than in Głogów. Hg is one of the elements (together with Pb, Cd, and As) which do not play a beneficial role in plant growth [37]. Hence, it can be supposed that even very low concentrations of these elements will have adverse effects on the plants’ growth. In the paper by Kibra, 2008 [41], the results showed that a soil contaminated with 1 mg·kg^−1^ of Hg led to a significant reduction in height of rice. In our study in Legnica, the Hg concentration was even higher than 1 mg·kg^−1^, which supposedly led to such a high toxicity effect. On the other hand, phytotoxicity tests in Siechnice and Oława indicated a more negative impact on plants in Oława than in Siechnice (Figure 3). The result is not very surprising due to the fact that in Oława, much higher amounts of Pb and Zn were observed. An excessive amount of Zn is known to adversely affect plant growth [42], whereas too large an amount of Pb in soil is thought to slow down the seed germination and plant development [43]. Both these elements in Oława exceeded the permissible concentrations (Table S5); therefore, such a negative impact on the plants is not surprising. 

Similar to what we obtained for soil analysis in Legnica, it was also found in a previous study [23,44] that the air is highly contaminated by Cu, and, as seen by the calculated contamination factor, the contamination was considered severe [23]. The contamination of the air by Cu was also noted for Głogów [45,46]. This is a confirmation of the serious problem with Cu-contamination in this region, as in our study, very high concentrations of Cu was found in the soil. The occurrence of As in both these studies is also not surprising due to the fact that Cu-smelting areas also deal with the problem of air contamination by As. Clearly, such air contamination significantly influences the indoor air quality. Air exchange between indoor and outdoor environments occur through ventilation and leaks in the buildings (infiltration), which may significantly influence the human health [47].

### 6.3. Health Risk Assessment

Given the results that we obtained, we wanted to explore further to check the possible impact of the PTEs on the human health. The calculations showed that in the Legnica and Głogów Copper Smelters, as well as in the Siechnice Smelter, the non-carcinogenic risk for human health was not observed. However, in one of the studied areas, i.e., the “Oława” Smelter, a possible health risk was noted, especially connected with the Pb contamination for children by ingestion pathway. This is very disturbing because especially small children show a tendency to hand and finger sucking which is considered to be one of the main exposure pathways of soil metals by children [48]. The exposure to the elevated levels of Pb is very dangerous as it can cause severe damage to the brain and kidneys, not only to children but also to adults, and it can ultimately result in death [49].

In terms of carcinogenic risk assessment, all of the areas we analysed revealed high risk level for selected elements (i.e., Siechnice (Cr, Pb), Głogów (As), Legnica (As), and Oława (Pb)). All these sampling points, located close to smelter areas, seem to be in a worse condition when compared with the studies from other parts of world, i.e., Romania [50] (where the highest risk was observed for Cd), Pakistan (where carcinogenic risk existed only in the case of Cr for children [51]), or China [52] (where most of the ECR values were at an acceptable level). In our study, the worst situation with ECR was observed for Cr in Siechnice (Table 2). However, when considering the concentration of this element, we can note that it does not really exceed the norm by much. This phenomenon can be explained by the fact that Cr is considered to be not only a toxic metal, but also one of the most carcinogenic metals toward cells [53]. Hence, even concentrations only slightly exceeding the limit can lead to serious consequences.

Presented cases prove that the mining and ore processing industry still has a critical impact on the environment and is able to produce large-scale changes in the environment. PTEs’ presence combined with runoff erosion of tailings and waste production and PM emission from smelters, are the sources of the environmental problems. Poor environmental practices and old technologies applied in the past resulted in large-scale contamination where polluted soils and plants serve as reservoir for pollution dispersion. Most terrifying are the elevated amounts of PTEs which can be also dangerous to human health. It is crucial to study past environmental burdens in order prevent contamination dispersion in the future.

## 7. Conclusions

Our study describes the impact of mining and ore processing activities on the environment. Four study areas have been chosen in the Lower Silesia region in order to identify the most important PTEs responsible for contamination and their impact on plants and humans. According to the complex pollution assessment conducted in this study, it is apparent that that soils from the smelter areas in Poland are polluted by PTEs in varying degrees. For Siechnice, the most common element was Fe, then in descending order: Cr > Cu > Zn > Pb; for Oława, the order was: Zn > Pb > Cd; and for the Legnica and Głogów Copper Smelters, it was as follows: Cu > As > Hg. Despite the same order of the elements, higher toxicity on plants was observed in the Legnica Copper Smelter when compared with Głogów. In addition, the phytotoxicity tests indicated that the plants in the area of Oława were more negatively impacted than those from Siechnice. In the terms of human health, the non-carcinogenic levels are rather acceptable. Only soils collected in the area of the “Oława” Smelter revealed the presence of non-carcinogenic risk. However, all the samples from these four study areas showed the possibility of carcinogenic risk occurrence, and the highest ECR was noted in Siechnice. Therefore, some serious steps should be undertaken in order to reduce the environmental exposure risk connected with the occurrence of PTEs in the soils to protect vulnerable groups like children. Furthermore, if the soil is in bad condition we can also suppose that the air could be impacted as well—directly by the smelters or indirectly by ascent of the pollution particles from the ground.

The study that we conducted is crucial for understanding the impact of smelters on the environment and human health and to detect the long-term impacts and the consequences of environmental pollution for future generations.

## Figures and Tables

**Table 1:** The concentrations of heavy metals [mg·kg^−1^] in the vicinity of four studied smelters.

	Concentration [mg·kg^−1^]					
Studied Element	Siechnice					
	S0	S2	S3	S4		S5
Zn	84.84 ± 2.31	80.29 ± 4.10	68.91 ± 3.63	44.49 ± 17.81		81.09 ± 24.90
Cr	696.30 ± 21.14	222.03± 41.11	31.19 ± 9.10	153.15 ± 64.12		705.31 ± 29.84
Pb	17.32 ± 0.30	13.13 ± 0.14	25.76 ± 0.92	22.11 ± 3.62		44.33 ± 0.71
Fe	9539.59 ± 246.12	9973.82 ± 432.10	13,440.62 ± 342.63	6411.24 ± 180.22		8940.15 ± 67.45
Cu	77.56 ± 31.14	73.01 ± 7.12	81.34 ± 3.6	76.58 ± 29.1		88.48 ± 21.13
	Głogów		Legnica			
	G2-a	G3-a	L1	L2		L3
Cu	1316.78 ± 44.26	834.36 ± 4.12	1035.3 ± 67.67	580.91 ± 69.01		243.64 ± 34.14
Hg	0.443 ± 0.01	0.553 ± 0.05	4.07 ± 0.60	0.56 ± 0.02		0.47 ± 0.06
As	6.25 ± 1.23	-	7.6 ± 2.31	-		1.02 ± 0.04
	Oława					
	O1	O2	O3		O4	
Zn	2411.93 ± 27.16	917.53 ± 34.23	121.89 ± 34.18		23.67 ± 6.80	
Pb	1903.02 ± 48.13	434.91 ± 67.12	63.81 ± 12.98		28.89 ± 2.70	
Cd	4.97 ± 0.71	3.32 ±0.18	0.43 ± 0.12		0.27± 0.04	

- hyphen—denotes not studied.

**Table 2:** Excessive risk for cancer (ECR).

ECR		
**Sites**	**Cr (child)**	**Cr (adult)**
S0	2.20 × 10^0^	8.24 × 10^−1^
S2	7.01 × 10^−1^	2.63 × 10^−1^
S3	9.90 × 10^−2^	3.70 × 10^−2^
S4	4.80 × 10^−1^	1.80 × 10^−1^
S5	2.20 × 10^0^	8.40 × 10^−1^
	**Pb (child)**	**Pb (adult)**
S0	3.65 × 10^−4^	1.37 × 10^−4^
S2	2.76 × 10^−4^	1.03 × 10^−4^
S3	5.42 × 10^−4^	2.03 × 10^−4^
S4	4.65 × 10^−4^	1.70 × 10^−4^
S5	9.32 × 10^−4^	3.50 × 10^−4^
	**As (child)**	**As (adult)**
G2-a	7.10 × 10^−2^	2.65 × 10^−2^
	**Pb (child)**	**Pb (adult)**
O1	4.00 × 10^−2^	1.50 × 10^−2^
O2	9.00 × 10^−3^	3.00 × 10^−3^
O3	1.20 × 10^−3^	5.00 × 10^−4^
O4	5.89 × 10^−4^	2.00 × 10^−4^
	**As (child)**	**As (adult)**
L1	8.60 × 10^−2^	3.22 × 10^−2^
L3	1.15 × 10^−2^	4.33 × 10^−3^

**Table 3:** PTEs concentrations in other countries.

PTE Concentrations [mg·kg^−1^]									
Country	As	Cd	Cr	Cu	Pb	Zn	Fe	Hg	References
China	565.50	373.60	98.20	1050.50	3331.80	9743.90			[31]
Poland	59.08	2.92		913.33	86.67			0.13	[32]
China	126.41	12.69	86.18	87.10	369.50	530.41		0.51	[33]
Kosovo	9.80	7.70	160.00	44.00	220.00	280.00	2800.00	0.25	[34]
South Korea	90.00	3.30	110.00	72.00	1300.00	520.00	3400.00	0.49	[35]
Serbia	16.90	3.20		154.80	411.70	102.60			[36]
Siechnice, Poland		12.32		20.06	781.91	1062.98			This work
Głogów, Poland			361.59	79.40	24.52	71.92	9661.08		This work
Oława, Poland				1075.60				0.50	This work
Legnica, Poland		2.20			597.00	860.00			This work

## Data Availability

Not applicable.

## Supplementary Materials

The following supporting information can be downloaded at: https://www.mdpi.com/article/10.3390/ijerph191912634/s1. Table S1. Description of sampling sites. Table S2. The value for parameters for calculation of exposure dose. Table S3. The values of the reference doses (RfD). Table S4. Results of one-way ANOVA for studied sites within smelters. Table S5. The permissible concentrations of potentially toxic elements in the soil. Table S6. Average Daily Dose (ADD) values for mercury, copper and arsenic—Legnica. Table S7. Hazard Quotient (HQ) and Hazard Index values for mercury, copper and arsenic—Legnica. Table S8. Average Daily Dose (ADD), Hazard Quotient (HQ) and Hazard Index(HI) values for mercury, copper and arsenic—Glogow. Table S9. Average Daily Dose (ADD) for zinc, lead and cadmium—Oława. Table S10. Values of Hazard Quotient (HQ) and Hazard Index (HI) for zinc, lead and cadmium—Oława. Table S11. Average Daily Dose (ADD) values for zinc, copper, chromium, iron and lead—Siechnice. Table S12. Hazard Quotient (HQ) and Hazard Index (HI) for zinc, copper, chromium, iron and lead—Siechnice.
