# Supplementary material for: The Comprehensive Health Risk Assessment of Polish Smelters with Ecotoxicological Studies
Source: Int J Environ Res Public Health. 2022 Oct 3;19(19):12634. doi: 10.3390/ijerph191912634 (PMC9564705; doi:10.3390/ijerph191912634)
Supplement: Supplementary file 1 [file ijerph-19-12634-s001.zip › ijerph-1898995-supplementary.pdf]

Supplementary material

Table S1. Description of sampling sites.

Site	Location	Description	Coordinates
**S0**	Siechnice	The site is about 0.7 km from the slag heap in the vicinity of the main road in direction to Wrocław (Grafitowa street). Companies dealing with laser cutting, the electrical industry and car service are located nearby.	51°04’47.1 "N 17°12’48.72"E
**S2**	Siechnice	This sampling site was located 100 m from the southern side of slag heap, along the eastern ring road of Wrocław. .	51°02'28.0"N 17°07'59.4"E
**S3**	Siechnice	This point was also located in the southern part of heap, about 40 m, near the eastern bypass of Wrocław.	51°02'28.5"N 17°08'07.7"E
**S4**	Siechnice	The sampling point was situated 30 m from the southern side of the heap, also near the eastern bypass of Wrocław.	51°02'29.2"N 17°08'13.2"E
**S5**	Siechnice	The sampling site located on the edge of the heap on the south-eastern side, about 100 m from the eastern bypass of Wrocław and 160 m from residential buildings.	51°02'30.2"N 17°08'16.7"E
**S1-b**	Siechnice	The site located 0.4 km from the slag heap, next to the drainage ditch. In close proximity to the place of collection, there was a street with significant traffic due to the vicinity of the cement manufacturing plant.	51° 2' 31.476" N 17° 7' 47.08" E
**S2-b**	Siechnice	This point was situated 0.3 km from the slag heap, next to the drainage ditch, too. Soil sampling was in the immediate vicinity of the residential houses.	51° 2' 22.931" N 17° 8' 2.456" E
**S3-b**	Siechnice	This site was in a meadow, about 0.2 km from the top of the heap. There were fruit trees in close proximity. The meadow is away from communication routes, however the company TOI TOI Polska Sp. z o.o. is located nearby, probably not having a significant impact on the adjacent meadow.	51° 2' 38.947" N 17° 8' 13.815" E

**S4-b**	Siechnice	This site is approx. 0.1 km from the top of the slag heap. The works of the waste processing plant are carried out directly at the site of soil sampling. Within a radius of about 150 m around the heap, the area is heavily transformed not only by the movement of excavators, but also by contamination of the soil with waste.	51° 2' 33.431" N<br>17° 8' 2.157" E
**G2-a**	Głogów	in The area of sampling is a meadow, in the leeward side from the smelter, in a potential stream of pollutants from the Głogów smelter. It was an open, flat meadow with single trees.	51°6’79.388" N<br>16°0’08.880" E
**G3-a**	Głogów	The point was located in the leeward side from the smelter, in the potential pollution stream of the Głogów smelter. It was in the vicinity of the forest and vegetation of height up to 1 m.	51°6’77.380" N<br>16°0’08.623" E
**G1-b**	Głogów	The point was situated more than 2.2 km north-west from the smelter. The point was classified as wasteland, i.e. areas of land which have lost their value in use as a result of industrial activity. These are lands that have been degraded by the Głogów Copper Smelter.	51°6’96.253" N<br>15°9’64.539" E
**G2-b**	Głogów	This site was located more than 1 km from the smelter to the south. The point was classified as wasteland, i.e. areas of land which have lost their value in use as a result of industrial activity. These are lands that have been degraded by the Głogów Copper Smelter.	51°6’81.585" N<br>15°9’81.681" E
**G3-b**	Głogów	The point was located less than 0.6 km from the smelter. The area was classified as wasteland, i.e. areas of land which have lost their value in use as a result of industrial activity. These are lands that have been degraded by the Głogów Copper Smelter.	51°6’84.511" N<br>16°0’00.636" E
**O1**	Oława	The point was situated in the immediate vicinity of the smelter, approximately 0.2 km from the main pollutant emitter (chimney), in the west/north-west of the smelter.	50°9’32.642 " N<br>17°3’01.220" E
**O2**	Oława	The area of sampling is situated 0.4 km from the smelter, in an area of a garden next to a residential building, in west/north-west of the smelter. In the neighbourhood there is a supermarket, built on the site of a former sawmill..	50°9’38.023" N<br>17°3’01.914" E

**O3**	Oława	The point was located 0.9 km from the pollutant emitter, in the meadow tin the west/north-west of the smelter.	50°9’33.305" N<br>17°3’12.698" E
**O4**	Oława	The point was located on an island on the river, about 1.5 km from the smelter. The site was also situated in the west/north-west of the smelter.	51°1’79.824" N<br>16°1’04.339" E
**L1**	Legnica	The site was situated in the leeward side, in the immediate vicinity of the main emitter, approximately 0.3 km away. There was no natural barrier separating the area from the emitter. There were no major communication routes or paved roads near this sampling point.	51°1’86.485" N<br>16°1’13.424" E
**L2**	Legnica	The point was located in the leeward side from the smelter, near the slag heap, at a distance of about 0.1 km. The area was separated from pollution source with a thin strip of trees and shrubs	51°1’79.918"N<br>16°1’16.544" E
**L3**	Legnica	The point was located in the leeward side from the smelter, near the copper sludge post-flotation tank, at a distance of about 0.05 km. The area was fenced off with thick trees and shrubs.	51°1’79.824" N<br>16°1’04.339" E

Table S2. The value for parameters for calculation of exposure dose .

Parameter	Adults	Children
ingR	200 mg/d	100 mg/d
EF	180 d/year	180 d/year
ED	70 year	6 year
AT	70*365 d	6*365 d
BW	70 kg	15 kg
inhR	20 m^3/d	7.6 m^3/d
PEF	$1.39*10^9$ m^3/kg	$1.39*10^9$ m^3/kg
ABS	0.001	0.001

SL	0.7 $mg/cm^2 \cdot d$	0.2 $mg/cm^2 \cdot d$
SA	5700 cm^2	2800 cm^2

Table S3. The values of the reference doses (RfD)

ng/kg*d	RfDing	RfDinh	RfDderm
Zn	$3.00*10^{5}$	$3.00*10^{5}$	$6.00*10^{4}$
Cr	$3.00*10^{3}$	$2.86*10^{1}$	$6.00*10^{1}$
Cu	$4.00*10^{4}$	$4.00*10^{4}$	$1.20*10^{4}$
Fe	$7.00*10^{1}$	$7.00*10^{1}$	$7.40*10^{1}$
Pb	$1.40*10^{3}$	$3.50*10^{3}$	$5.20*10^{2}$
Cd	$1.00*10^{3}$	$1.00*10^{3}$	$1.00*10^{1}$
As	$3.00*10^{2}$	-	$3.00*10^{2}$
Hg	$3.00*10^{4}$	$8.57*10^{5}$	$2.10*10^{5}$

Table S4. Results of one- way ANOVA for studied sites within smelters.

Element	F value	Observed p value
Siechnice		
Zn	43.5	0.0000003*
Cr	4754.8	0.0000000*
Pb	79.5	0.0000000*
Fe	226.03	0.0000000*
Cu	9.8	0.002*
Głogów		
Cu	38.12	0.002*
Hg	1.96	0.19
As	31.5	0.0005*
Legnica		
Cu	1.73	0.2
Hg	6.63	0.01*
As	16.1	0.001*
Oława		
Zn	845.2	0.0000000*
Pb	39.1	0.00004*
Cd	376.8	0.0000000*

*Significant differences among samples collected at different sites within areas of studied smelters (p values < 0.05).

Table S5. The permissible concentrations of potentially toxic elements in the soil (DZIENNIK *USTAW RZECZYPOSPOLITEJ POLSKIEJ*, n.d.)

Elements	Concentrations [mg/kg]
**As**	20
**Pb**	250
**Cd**	3
**Cr**	300
**Cu**	150
**Hg**	4
**Zn**	500

Table S6. Average Daily Dose (ADD) values for mercury, copper and arsenic – Legnica.

	Hg Adults [mg/kg]			Hg Children [mg/kg]		
Sites	ADDing	ADDinh	ADDderm	ADDing	ADDinh	ADDderm
L1	$5.73 * 10^{0}$	$4.13*10^{-4}$	$1.14*10^{-1}$	$1.34 * 10^{1}$	$7.32*10^{-4}$	$7.49*10^{-2}$
L2	$7.8 * 10^{-1}$	$5.68*10^{-5}$	$1.57*10^{-2}$	$1.84 * 10^{0}$	$1.01*10^{-4}$	$1.03*10^{-2}$
L3	$6.6 * 10^{-1}$	$4.76*10^{-5}$	$1.32*10^{-2}$	$1.54 * 10^{0}$	$8.45*10^{-5}$	$8.65*10^{-3}$
	**Cu Adults [mg/kg]**			**Cu Children [mg/kg]**		
Sites	ADDing	ADDinh	ADDderm	ADDing	ADDinh	ADDderm
L1	$1.46 * 10^{3}$	$1 * 10^{-1}$	$2.91 * 10^{1}$	$3.40 * 10^{3}$	$1.8 * 10^{-1}$	$1.90 * 10^{1}$
L2	$8.17 * 10^{2}$	$5.9 * 10^{-2}$	$1.63 * 10^{1}$	$1.90 * 10^{3}$	$1 * 10^{-1}$	$1.07 * 10^{1}$
L3	$3.38 * 10^{2}$	$2.4 * 10^{-1}$	$6.74 * 10^{0}$	$7.89 * 10^{2}$	$4 * 10^{-2}$	$4.41 * 10^{0}$
	**As Adults [mg/kg]**			**As Children [mg/kg]**		
Sites	ADDing	ADDinh	ADDderm	ADDing	ADDinh	ADDderm
L1	$1.07 * 10^{1}$	$7.70*10^{-4}$	$2.14*10^{-1}$	$1.12*10^{-2}$	-	$1.40*10^{-1}$
L2	-	-	-	-	-	-
L3	$1.43 * 10^{0}$	$1.03*10^{-4}$	$2.87*10^{-2}$	$3.35 * 10^{0}$	-	$1 * 10^{-2}$

Table S7. Hazard Quotient (HQ) and Hazard Index values for mercury, copper and arsenic – Legnica.

**HQing**	**Hg (Adults)**	**Hg (Children)**	**Cu (Adults)**	**Cu (Children)**	**As (Adults)**	**As (Children)**
**L1**	$1.91*10^{-4}$	$4.46*10^{-4}$	$3.65*10^{-2}$	$8.51*10^{-2}$	$3.57*10^{-2}$	$8.51*10^{-2}$
**L2**	$2.63*10^{-5}$	$6.14*10^{-5}$	$2.04*10^{-2}$	$4.77*10^{-2}$	-	-
**L3**	$2.21*10^{-5}$	$5.15*10^{-5}$	$8.45*10^{-3}$	$1.97*10^{-2}$	$4.79*10^{-3}$	$1 * 10^{-2}$
**HQinh**	**Hg (Adults)**	**Hg (Children)**	**Cu (Adults)**	**Cu (Children)**	**As (Adults)**	**As (Children)**
**L1**	$4.81*10^{-10}$	$8.54*10^{-10}$	$2.62*10^{-6}$	$4.65*10^{-6}$	-	-
**L2**	$6.62*10^{-11}$	$1.17*10^{-10}$	$6.62*10^{-11}$	$2.61*10^{-6}$	-	-
**L3**	$5.56*10^{-11}$	$9.86*10^{-11}$	$6.08*10^{-6}$	$1.08*10^{-6}$	-	-
**HQderm**	**Hg (Adults)**	**Hg (Children)**	**Cu (Adults)**	**Cu (Children)**	**As (Adults)**	**As (Children)**
**L1**	$5.45*10^{-7}$	$3.57*10^{-7}$	$2.42*10^{-3}$	$1.59*10^{-3}$	$7.12*10^{-4}$	$4.66*10^{-4}$
**L2**	$7.49*10^{-8}$	$4.91*10^{-8}$	$7.50*10^{-8}$	$8.90*10^{-4}$	-	-
**L3**	$6.29*10^{-8}$	$4.12*10^{-8}$	$5.62*10^{-4}$	$3.68*10^{-4}$	$9.56*10^{-5}$	$6.26*10^{-5}$
**HI**	**Hg (Adults)**	**Hg (Children)**	**Cu (Adults)**	**Cu (Children)**	**As (Adults)**	**As (Children)**
**L1**	$1.92*10^{-4}$	$4.46*10^{-4}$	$3.89*10^{-2}$	$8.67*10^{-2}$	$3.64*10^{-2}$	$8 * 10^{-2}$
**L2**	$2.64*10^{-5}$	$6.14*10^{-5}$	$2.64*10^{-5}$	$4.86*10^{-2}$	-	-
**L3**	$2.21*10^{-5}$	$5.15*10^{-5}$	$9.02*10^{-3}$	$2.01*10^{-2}$	$4.89*10^{-3}$	$1.12*10^{-2}$

Table S8. Average Daily Dose (ADD), Hazard Quotient (HQ) and Hazard Index(HI) values for mercury, copper and arsenic – Glogow.

	**Hg Adults[mg/kg]**				**Hg Children[mg/kg]**			
Sites	ADDing	ADDinh	ADDderm		ADDing	ADDinh		ADDderm
**G2-a**	$6.2 * 10^{-1}$	$4.49*10^{-5}$	$1.25*10^{-2}$		$1.46 * 10^{0}$	$7.96*10^{-5}$		$8.16*10^{-3}$
**G3-a**	$7.5 * 10^{-1}$	$5.40*10^{-5}$	$1.50*10^{-2}$		$1.75 * 10^{0}$	$9.58*10^{-5}$		$9.81*10^{-3}$
	HQing	HQinh	HQderm	HI	HQing	HQinh	HQderm	HI
**G2-a**	$2.08*10^{-5}$	$5.24*10^{-11}$	$5.93*10^{-8}$	$2.09*10^{-5}$	$4.85*10^{-5}$	$9.29*10^{-11}$	$3.88*10^{-8}$	$4.86*10^{-5}$
**G3-a**	2.50*10-5	$6.30*10^{-11}$	$7.13*10^{-8}$	$2.51*10^{-5}$	$5.84*10^{-5}$	$1.12*10^{-10}$	$4.67*10^{-8}$	$5.85*10^{-5}$
	**Cu Adults[mg/kg]**				**Cu Children[mg/kg]**			
	ADDing	ADDinh	ADDderm		ADDing	ADDinh		ADDderm
**G2-a**	$1.85 * 10^{3}$	$1.3 * 10^{-1}$	$3.70 * 10^{1}$		$4.33 * 10^{3}$	$2.3 * 10^{-1}$		$2.42 * 10^{1}$
**G3-a**	$1.17 * 10^{3}$	$8.4 * 10^{-1}$	$2.34 * 10^{1}$		$2.74 * 10^{3}$	$1.4 * 10^{-1}$		$1.53 * 10^{1}$
	HQing	HQinh	HQderm	HI	HQing	HQinh	HQderm	HI
**G2-a**	$4 * 10^{-2}$	$3.34*10^{-6}$	$3.08*10^{-3}$	$4.95*10^{-2}$	$1.08*10^{-1}$	$5.92*10^{-6}$	$2.02*10^{-3}$	$1.10*10^{-1}$
**G3-a**	$2.94*10^{-2}$	$2.11*10^{-5}$	$1.95*10^{-3}$	$3.14*10^{-2}$	$6.86*10^{-2}$	$3.75*10^{-6}$	$1.28*10^{-3}$	$6.99*10^{-2}$
	**As Adults[mg/kg]**				**As Children[mg/kg]**			
	ADDing	ADDinh	ADDderm		ADDing	ADDinh		ADDderm
**G2-a**	$8.80 * 10^{0}$	$6.34*10^{-4}$	$1.76*10^{-1}$		$2.05 * 10^{1}$	$1.12*10^{-3}$		$1.15*10^{-1}$
	HQing	HQinh	HQderm	HI	HQing	HQinh	HQderm	HI
**G2-a**	$2.94*10^{-2}$	-	$5.86*10^{-4}$	$2.99*10^{-2}$	$6.85*10^{-2}$	-	$3.84*10^{-4}$	$6.89*10^{-2}$

Table S9. Average Daily Dose (ADD) for zinc, lead and cadmium - Oława

	**Zn Adults [mg/kg]**			**Zn Children [mg/kg]**		
Sites	ADDing	ADDinh	ADDderm	ADDing	ADDinh	ADDderm
O1	$3.38 * 10^{3}$	$2.4 * 10^{-1}$	$6.75 * 10^{1}$	$7.89 * 10^{3}$	$4.3 * 10^{-1}$	$4.42 * 10^{1}$
O2	$1.27 * 10^{3}$	$9 * 10^{-2}$	$2.53 * 10^{1}$	$2.96 * 10^{3}$	$1.6 * 10^{-1}$	$1.66 * 10^{1}$
O3	$1.69 * 10^{2}$	$1.2 * 10^{-2}$	$3.37 * 10^{0}$	$3.95 * 10^{2}$	$2 * 10^{-2}$	$2.20 * 10^{0}$
O4	$2.82 * 10^{1}$	$2.03*10^{-3}$	$5.6 * 10^{-1}$	$6.57 * 10^{1}$	$3.60*10^{-3}$	$3.6 * 10^{-1}$
	**Pb Adults [mg/kg]**			**Pb Children [mg/kg]**		
Sites	ADDing	ADDinh	ADDderm	ADDing	ADDinh	ADDderm
O1	$2.68 * 10^{3}$	$1.9 * 10^{-1}$	$5.34 * 10^{1}$	$6.24 * 10^{3}$	$3.4 * 10^{-1}$	$3.50 * 10^{1}$
O2	$6.05 * 10^{2}$	$4 * 10^{-2}$	$1.21 * 10^{1}$	$1.41 * 10^{3}$	$7 * 10^{-2}$	$7.91 * 10^{0}$
O3	$8.45 * 10^{1}$	$6.08*10^{-3}$	$1.68 * 10^{0}$	$1.97 * 10^{2}$	$1 * 10^{-2}$	$1.10 * 10^{0}$
O4	$3.95 * 10^{1}$	$2.84*10^{-3}$	$7.8 * 10^{-1}$	$9.20 * 10^{1}$	$5.03*10^{-3}$	$5.1 * 10^{-1}$
	**Cd Adults [mg/kg]**			**Cd Children [mg/kg]**		
Sites	ADDing	ADDinh	ADDderm	ADDing	ADDinh	ADDderm
O1	$6.90 * 10^{0}$	$4.97*10^{-4}$	$1.3 * 10^{-1}$	$1.61 * 10^{1}$	$8.81*10^{-4}$	$9 * 10^{-2}$
O2	$4.65 * 10^{0}$	$3.35*10^{-4}$	$9 * 10^{-2}$	$1.08 * 10^{1}$	$5.93*10^{-4}$	$6 * 10^{-2}$
O3	$5.6 * 10^{-1}$	$4.05*10^{-5}$	$1 * 10^{-2}$	$1.31 * 10^{0}$	$7.19*10^{-5}$	$7.36*10^{-3}$

O4	$2.8 * 10^{-1}$	$2.03*10^{-5}$	$5.62*10^{-3}$	$6.5 * 10^{-1}$	$3.60*10^{-5}$	$3.68*10^{-3}$

Table S10. Values of Hazard Quotient (HQ) and Hazard Index (HI) for zinc, lead and cadmium - Oława

**HQ*ing***	**Zn (adults)**	**Zn (children)**	**Pb (adults)**	**Pb (children)**	**Cd (adults)**	**Cd (children)**
O1	$1.13*10^{-2}$	$2.63*10^{-2}$	$1.91*10^{0}$	$4.46*10^{0}$	$6.90*10^{-3}$	$1.61*10^{-2}$
O2	$4.23*10^{-3}$	$9.86*10^{-3}$	$4.33*10^{-1}$	$1.01*10^{0}$	$4.65*10^{-3}$	$1.08*10^{-2}$
O3	$5.64*10^{-4}$	$1.32*10^{-3}$	$6.04*10^{-2}$	$1.41*10^{-1}$	$5.64*10^{-4}$	$1.32*10^{-3}$
O4	$9.39*10^{-5}$	$2.19*10^{-4}$	$2.82*10^{-2}$	$6.58*10^{-2}$	$2.82*10^{-4}$	$6.58*10^{-4}$
**HQ*inh***	**Zn (adults)**	**Zn (children)**	**Pb (adults)**	**Pb (children)**	**Cd (adults)**	**Cd (children)**
O1	$8.11*10^{-7}$	$1.44*10^{-6}$	$5.47*10^{-5}$	$9.70*10^{-5}$	$4.97*10^{-7}$	$8.81*10^{-7}$
O2	$3.04*10^{-7}$	$5.39*10^{-7}$	$1.24*10^{-5}$	$2.20*10^{-5}$	$3.35*10^{-7}$	$5.93*10^{-7}$
O3	$4.05*10^{-8}$	$7.19*10^{-8}$	$1.73*10^{-6}$	$3.06*10^{-6}$	$4.05*10^{-8}$	$7.19*10^{-8}$
O4	$6.76*10^{-9}$	$1.20*10^{-8}$	$8.06*10^{-7}$	$1.43*10^{-6}$	$2.03*10^{-8}$	$3.60*10^{-8}$
**HQderm**	**Zn (adults)**	**Zn (children)**	**Pb (adults)**	**Pb (children)**	**Cd (adults)**	**Cd (children)**
O1	$1.12*10^{-2}$	$7.36*10^{-3}$	$1.03*10^{-1}$	$6.73*10^{-2}$	$1.38*10^{-2}$	$9.02*10^{-3}$
O2	$4.22*10^{-3}$	$2.76*10^{-3}$	$2.32*10^{-2}$	$1.52*10^{-2}$	$9.28*10^{-3}$	$6.08*10^{-3}$
O3	$5.62*10^{-4}$	$3.68*10^{-4}$	$3.24*10^{-3}$	$2.12*10^{-3}$	$1.12*10^{-3}$	$7.36*10^{-4}$
O4	$9.37*10^{-5}$	$6.14*10^{-5}$	$1.51*10^{-3}$	$9.91*10^{-4}$	$5.62*10^{-4}$	$3.68*10^{-4}$
**HI**	**Zn (adults)**	**Zn (children)**	**Pb (adults)**	**Pb (children)**	**Cd (adults)**	**Cd (children)**
O1	$2.25*10^{-2}$	$3.37*10^{-2}$	$2.01*10^{0}$	$4.53*10^{0}$	$2.07*10^{-2}$	$2.51*10^{-2}$
O2	$8.44*10^{-3}$	$1.26*10^{-2}$	$4.56*10^{-1}$	$1.03*10^{0}$	$1.39*10^{-2}$	$1.69*10^{-2}$
O3	$1.13*10^{-3}$	$1.68*10^{-3}$	$6.36*10^{-2}$	$1.43*10^{-1}$	$1.69*10^{-3}$	$2.05*10^{-3}$
O4	$1.88*10^{-4}$	$2.81*10^{-4}$	$2.97*10^{-2}$	$6.67*10^{-2}$	$8.44*10^{-4}$	$1.03*10^{-3}$

Table S11. Average Daily Dose (ADD) values for zinc, copper, chromium, iron and lead - Siechnice.

	**Zn Adults [mg/kg]**			**Zn Children [mg/kg]**		
	ADDing	ADDinh	ADDderm	ADDing	ADDinh	ADDderm
S0	$1.20*10^{2}$	$8.60*10^{-3}$	$2.38*10^{0}$	$2.79*10^{2}$	$1.53*10^{-2}$	$1.56*10^{0}$
S2	$1.13*10^{2}$	$8.14*10^{-3}$	$2.26*10^{0}$	$2.64*10^{2}$	$1.44*10^{-2}$	$1.48*10^{0}$
S3	$9.71*10^{1}$	$6.98*10^{-3}$	$1.94*10^{0}$	$2.27*10^{2}$	$1.24*10^{-2}$	$1.27*10^{0}$
S4	$6.27*10^{1}$	$4.51*10^{-3}$	$1.25*10^{0}$	$1.46*10^{2}$	$8.00*10^{-3}$	$8.20*10^{-1}$
S5	$1.14*10^{2}$	$8.22*10^{-3}$	$2.28*10^{0}$	$2.67*10^{2}$	$1.46*10^{-2}$	$1.49*10^{0}$
	**Cu Adults [mg/kg]**			**Cu Children [mg/kg]**		
Sites	ADDing	ADDinh	ADDderm	ADDing	ADDinh	ADDderm
S0	$1.09*10^{2}$	$7.86*10^{-3}$	$2.18*10^{0}$	$2.55*10^{2}$	$1.39*10^{-2}$	$1.43*10^{0}$
S2	$1.03*10^{2}$	$7.40*10^{-3}$	$2.05*10^{0}$	$2.40*10^{2}$	$1.31*10^{-2}$	$1.34*10^{0}$
S3	$1.15*10^{2}$	$8.25*10^{-3}$	$2.29*10^{0}$	$2.67*10^{2}$	$1.46*10^{-2}$	$1.50*10^{0}$
S4	$1.08*10^{2}$	$7.76*10^{-3}$	$2.15*10^{0}$	$2.52*10^{2}$	$1.38*10^{-2}$	$1.41*10^{0}$
S5	$1.25*10^{2}$	$8.97*10^{-3}$	$2.49*10^{0}$	$2.91*10^{2}$	$1.59*10^{-2}$	$1.63*10^{0}$
	**Cr Adults [mg/kg]**			**Cr Children [mg/kg]**		

Sites	ADDing	ADDinh	ADDderm	ADDing	ADDinh	ADDderm
S0	$9.81*10^{2}$	$7.06*10^{-2}$	$1.98*10^{1}$	$2.29*10^{3}$	$1.30*10^{-1}$	$1.28*10^{1}$
S2	$3.13*10^{2}$	$2.25*10^{-2}$	$6.24*10^{0}$	$7.30*10^{2}$	$4.00*10^{-2}$	$4.09*10^{0}$
S3	$4.40*10^{1}$	$3.16*10^{-3}$	$8.80*10^{-1}$	$1.03*10^{2}$	$5.61*10^{-3}$	$5.70*10^{-1}$
S4	$2.16*10^{2}$	$1.55*10^{-2}$	$4.30*10^{0}$	$5.03*10^{2}$	$3.00*10^{-2}$	$2.82*10^{0}$
S5	$9.94*10^{2}$	$7.15*10^{-2}$	$1.98*10^{1}$	$2.32*10^{3}$	$1.30*10^{-1}$	$1.30*10^{1}$
	**Fe Adults[mg/kg]**			**Fe Children [mg/kg]**		
Sites	ADDing	ADDinh	ADDderm	ADDing	ADDinh	ADDderm
S0	$1.34*10^{4}$	$9.70*10^{-1}$	2.68*102	$3.14*10^{4}$	$1.71*10^{0}$	$1.76*10^{2}$
S2	$1.41*10^{4}$	$1.01*10^{0}$	$2.80*10^{2}$	$3.28*10^{4}$	$1.79*10^{0}$	$1.84*10^{2}$
S3	$1.89*10^{4}$	$1.36*10^{0}$	$3.78*10^{2}$	$4.42*10^{4}$	$2.42*10^{0}$	$2.47*10^{2}$
S4	$9.03*10^{3}$	$6.50*10^{-1}$	$1.80*10^{2}$	$2.11*10^{4}$	$1.15*10^{0}$	$1.18*10^{2}$
S5	$1.26*10^{4}$	$9.10*10^{-1}$	$2.51*10^{2}$	$2.94*10^{4}$	$1.61*10^{0}$	$1.65*10^{2}$
	**Pb Adults[mg/kg]**			**Pb Children [mg/kg]**		
Sites	ADDing	ADDinh	ADDderm	ADDing	ADDinh	ADDderm
S0	$2.44*10^{1}$	$1.76*10^{-3}$	$4.90*10^{-1}$	$5.70*10^{1}$	$3.11*10^{-3}$	$3.20*10^{-1}$
S2	$1.85*10^{1}$	$1.33*10^{-3}$	$3.70*10^{-1}$	$4.31*10^{1}$	$2.36*10^{-3}$	$2.40*10^{-1}$
S3	$3.63*10^{1}$	$2.61*10^{-3}$	$7.20*10^{-1}$	$8.47*10^{1}$	$4.63*10^{-3}$	$4.70*10^{-1}$
S4	$3.12*10^{1}$	$2.24*10^{-3}$	$6.20*10^{-1}$	$7.27*10^{1}$	$3.97*10^{-3}$	$4.10*10^{-1}$
S5	$6.24*10^{1}$	$4.49*10^{-3}$	$1.25*10^{0}$	$1.46*10^{2}$	$7.96*10^{-3}$	$8.20*10^{-1}$

Table S12. Hazard Quotient (HQ) and Hazard Index (HI) for zinc, copper, chromium, iron and lead - Siechnice

**HQ*ing***	**Zn (adults)**	**Zn (children)**	**Pb (adults)**	**Pb (children)**	**Cu (adults)**	**Cu (children)**	**Fe (adults)**	**Fe (children)**	**Cr (adults)**	**Cr (children)**
S0	$3.98*10^{-4}$	$9.30*10^{-4}$	$1.71*10^{-2}$	$3.98*10^{-2}$	$2.73*10^{-3}$	$6.37*10^{-3}$	$1.92*10^{-2}$	$4.48*10^{-2}$	$3.27*10^{-1}$	$7.63*10^{-1}$
S2	$3.77*10^{-4}$	$8.80*10^{-4}$	$1.29*10^{-2}$	$3.01*10^{-2}$	$2.57*10^{-3}$	$6.00*10^{-3}$	$2.01*10^{-2}$	$4.68*10^{-2}$	$1.04*10^{-1}$	$2.43*10^{-1}$
S3	$3.24*10^{-4}$	$7.55*10^{-4}$	$2.54*10^{-2}$	$5.92*10^{-2}$	$2.87*10^{-3}$	$6.69*10^{-3}$	$2.71*10^{-2}$	$6.31*10^{-2}$	$1.47*10^{-2}$	$3.42*10^{-2}$
S4	$2.09*10^{-4}$	$4.88*10^{-4}$	$2.18*10^{-2}$	$5.08*10^{-2}$	$2.70*10^{-3}$	$6.29*10^{-3}$	$1.29*10^{-2}$	$3.01*10^{-2}$	$7.19*10^{-2}$	$1.68*10^{-1}$
S5	$3.81*10^{-4}$	$8.89*10^{-4}$	$4.37*10^{-2}$	$1.02*10^{-1}$	$3.12*10^{-3}$	$7.27*10^{-3}$	$1.80*10^{-2}$	$4.20*10^{-2}$	$3.31*10^{-1}$	$7.73*10^{-1}$
**HQ*inh***	**Zn (adults)**	**Zn (children)**	**Pb (adults)**	**Pb (children)**	**Cu (adults)**	**Cu (children)**	**Fe (adults)**	**Fe (children)**	**Cr (adults)**	**Cr children)**
S0	$2.87*10^{-8}$	$5.08*10^{-8}$	$4.99*10^{-7}$	$8.85*10^{-7}$	$1.97*10^{-7}$	$3.49*10^{-7}$	$1.38*10^{-6}$	$2.45*10^{-6}$	$2.47*10^{-3}$	$4.38*10^{-3}$
S2	$2.71*10^{-8}$	$4.81*10^{-8}$	$3.77*10^{-7}$	$6.69*10^{-7}$	$1.85*10^{-7}$	$3.28*10^{-7}$	$1.44*10^{-6}$	2.56*10-^{6}	$7.87*10^{-4}$	$1.40*10^{-3}$
S3	$2.33*10^{-8}$	$4.13*10^{-8}$	$7.42*10^{-7}$	$1.32*10^{-6}$	$2.06*10^{-7}$	$3.66*10^{-7}$	$1.95*10^{-6}$	$3.45*10^{-6}$	$1.11*10^{-4}$	$1.96*10^{-4}$
S4	$1.50*10^{-8}$	$2.67*10^{-8}$	$6.37*10^{-7}$	$1.13*10^{-6}$	$1.94*10^{-7}$	$3.44*10^{-7}$	$9.28*10^{-7}$	$1.65*10^{-6}$	$5.43*10^{-4}$	$9.63*10^{-4}$
S5	$2.74*10^{-8}$	$4.86*10^{-8}$	$1.28*10^{-6}$	$2.26*10^{-6}$	$2.24*10^{-7}$	$3.98*10^{-7}$	$1.29*10^{-6}$	$2.30*10^{-6}$	$2.50*10^{-3}$	$4.43*10^{-3}$
**HQ *derm***	**Zn (adults)**	**Zn (children)**	**Pb (adults)**	**Pb (children)**	**Cu (adults)**	**Cu (children)**	**Fe (adults)**	**Fe (children)**	**Cr (adults)**	**Cr children)**
S0	$3.97*10^{-5}$	$2.60*10^{-5}$	$9.29*10^{-4}$	$6.09*10^{-4}$	$1.82*10^{-4}$	$1.19*10^{-4}$	$3.63*10^{-4}$	$2.38*10^{-4}$	$6.52*10^{-3}$	$4.27*10^{-3}$
S2	$3.76*10^{-5}$	$2.46*10^{-5}$	$7.03*10^{-4}$	$4.60*10^{-4}$	$1.71*10^{-4}$	$1.12*10^{-4}$	$3.80*10^{-4}$	$2.49*10^{-4}$	$2.08*10^{-3}$	$1.36*10^{-3}$

S3	$3.23*10^{-5}$	$2.11*10^{-5}$	$1.38*10^{-3}$	$9.05*10^{-4}$	$1.91*10^{-4}$	$1.25*10^{-4}$	$5.12*10^{-4}$	$3.35*10^{-4}$	$2.92*10^{-4}$	$1.91*10^{-4}$
S4	$2.08*10^{-5}$	$1.37*10^{-5}$	$1.19*10^{-3}$	$7.77*10^{-4}$	$1.79*10^{-4}$	$1.17*10^{-4}$	$2.44*10^{-4}$	$1.60*10^{-4}$	$1.43*10^{-3}$	$9.40*10^{-4}$
S5	$3.80*10^{-5}$	$2.49*10^{-5}$	$2.38*10^{-3}$	$1.56*10^{-3}$	$2.07*10^{-4}$	$1.36*10^{-4}$	$3.41*10^{-4}$	$2.23*10^{-4}$	$6.61*10^{-3}$	$4.33*10^{-3}$
**HI**	**Zn (adults)**	**Zn (children)**	**Pb (adults)**	**Pb (children)**	**Cu (adults)**	**Cu (children)**	**Fe (adults)**	**Fe (children)**	**Cr (adults)**	**Cr children)**
S0	$4.38*10^{-4}$	$9.56*10^{-4}$	$1.80*10^{-2}$	$4.04*10^{-2}$	$2.91*10^{-3}$	$6.49*10^{-3}$	$1.96*10^{-2}$	$4.50*10^{-2}$	$3.36*10^{-1}$	$7.72*10^{-1}$
S2	$4.15*10^{-4}$	$9.05*10^{-4}$	$1.36*10^{-2}$	$3.06*10^{-2}$	$2.74*10^{-3}$	$6.11*10^{-3}$	$2.05*10^{-2}$	$4.71*10^{-2}$	$1.07*10^{-1}$	$2.46*10^{-1}$
S3	$3.56*10^{-4}$	$7.76*10^{-4}$	$2.68*10^{-2}$	$6.01*10^{-2}$	$3.06*10^{-3}$	$6.81*10^{-3}$	$2.76*10^{-2}$	$6.35*10^{-2}$	$1.51*10^{-2}$	$3.46*10^{-2}$
S4	$2.30*10^{-4}$	$5.01*10^{-4}$	$2.30*10^{-2}$	$5.16*10^{-2}$	$2.88*10^{-3}$	$6.41*10^{-3}$	$1.32*10^{-2}$	$3.03*10^{-2}$	$7.39*10^{-2}$	$1.70*10^{-1}$
S5	$4.19*10^{-4}$	$9.14*10^{-4}$	$4.60*10^{-2}$	$1.03*10^{-1}$	$3.32*10^{-3}$	$7.41*10^{-3}$	$1.83*10^{-2}$	$4.22*10^{-2}$	$3.40*10^{-1}$	$7.82*10^{-1}$
